# Concentration of fine needle aspirates similar to molecular method improves sensitivity of the diagnosis of tuberculous lymphadenitis in Addis Ababa, Ethiopia

**DOI:** 10.1186/s12879-017-2194-2

**Published:** 2017-01-14

**Authors:** Olifan Zewdie, Tamrat Abebe, Adane Mihret, Eyob Hirpa, Gobena Ameni

**Affiliations:** 1 Department of Medical Laboratory Sciences, College of Medical and Health Sciences, Wollega University, PO Box 395, Nekemte, Ethiopia; 2 Department of Microbiology, Immunology and Parasitology, School of Medicine, Addis Ababa University, Addis Ababa, Ethiopia; 3 School of Veterinary Medicine, Wollega University, Nekemte, Ethiopia; 4 Aklilu Lemma Institute of Pathobiology, Addis Ababa University, Addis Ababa, Ethiopia

**Keywords:** Smear concentration, PCR, TBLN, FNA, Addis Ababa

## Abstract

**Background:**

Tuberculous lymphadenitis (TBLN) diagnosis has been a true challenge solely by clinical evidence in developing countries, due to limited the diagnostic facility on hand. However, the availability and affordability of available diagnostic tools in resource-limited settings like Ethiopia necessitates the quest for other techniques with added value over direct Z-N microscopy. Therefore, we aimed at to assess whether the concentration of lymph node aspirate similarly improves the detection rate of tuberculous lymphadenitis or not.

**Materials and methods:**

A cross-sectional study design was conducted on 132 individual subjects presumptive for tuberculous lymphadenitis from February to October 2013 in Addis Ababa, Ethiopia. Fine needle aspirate (FNA) samples were collected from the cases and cultured on Löwenstein-Jensen (LJ) slants. Identification of species and strains of mycobacteria was made by region of difference (RD) based polymerase chain reaction (PCR). Data entry and statistical analyses were performed by SPSS version 20. The confidence level of 95% was used for statistical significance.

**Result:**

A total of 132 study subjects were included in our study. Of these 56.1% (74/132) were positive for *M. tuberculosis* on culture. The detection rate of direct smear microscopy and the concentration method were 29.5 and 65.2% respectively. The sensitivity of direct smear microscopy was 43.2%, for concentrated smear microscopy 94.5%, for PCR 93.2% and for cytomorphology 95.4%. The level of agreement of concentrated ZN smear microscopy was 0.62 which was very similar with kappa of 0.58 of molecular (PCR) technique. AFB positivity by the concentration method and molecular method was increased in caseous aspirates as compared to purulent and hemorrhagic aspirates though it was not statistically significant (*p*-value = 0.18) and (*p* = 0.62) respectively.

**Conclusion:**

The concentration of FNA (Fine Needle Aspirate) aspirates for acid-fast smear microscopy similarly improves the sensitivity of acid fast bacilli in diagnosing of TBLN.

## Background

*M. tuberculosis,* one of the most harmful human pathogens worldwide, causes considerable morbidity and mortality [1]. Tuberculosis is also a major public health problem in Ethiopia which ranks 7th among the 22 High Burden Tuberculosis Countries (HBTCs) and ranks 2nd EPTB cases globally [2]. The prevalence of all forms of TB is estimated at 261 per 100,000 population, leading to an annual mortality rate of 64 per 100,000 [3]. In Ethiopia, extra pulmonary tuberculosis (EPTB) accounted for 34.8% of TB cases with more than 80% of cases presenting as Tuberculosis lymphadenitis cases [4]. Even though early case detection and treatment is one of the pillars of the TB control program, extra pulmonary tuberculosis is a significant true challenge in its different clinical presentation. The challenges are due to the significant proportion of clinical samples, a low number of bacilli in EPTB specimens and their slow growth rate which reduce the sensitivity of conventional diagnostic methods [5].

Definitive diagnosis of tuberculous lymphadenitis is often difficult as most of the available techniques are low either in sensitivity or specificity as compared to culture. Clinical features, though indicative of tuberculous etiology, are not adequate for making a definitive diagnosis [6]. Fine-needle aspiration (FNA) has become a widely used diagnostic tool and it remains one of the most rapid and cost-effective diagnostic methods of tuberculous lymphadenitis but is characterized by low specificity [7]. The primary method for the diagnosis of tuberculosis is Ziehl Neelsen staining. Although the method is specific and rapid; the technique has low sensitivity in the detection of tubercle bacilli in various clinical specimens [8, 9]. The identification of tubercle bacilli by culture is required for the ultimate proof of mycobacterial infection. However, due to unavailability of laboratory equipment and safety procedures, the method is not practiced in resource-poor settings. [10, 11]. Nucleic acid methods in the specific target like Region of Difference are currently employed for rapid identification of mycobacteria from both cultured and uncultured clinical specimens [12].

Previous studies have shown that liquefaction of sputum and lymph node aspirate with some liquefaction chemical reagents and then concentrated by centrifugation before acid-fast staining significantly improves the sensitivity of smear microscopy [13, 14]. The aim of present study is to evaluate whether treatment with some chemical reagents followed by concentration by centrifugation of lymph node aspirate improves the sensitivity of smear microscopy similarly with compared to the molecular method.

## Methods

### Patient recruitment and study settings

This was a cross-sectional study conducted from February to October 2013 in Ethiopia at Tikur Anbessa Specialized Hospital (TASH) and Alem Tena Higher Clinic (ATHC). One hundred and thirty-two clinically suspected TBLN patients, who were referred to Pathology laboratory of TASH and ATHC and who gave their consent for the examination of fine needle aspirates (FNAs), were included in this study. Demographic data for all patients were collected using a pre structured questionnaire by trained clinical nurses. Patients who were below 18 years old and who were taking anti-TB treatment at the time of sample collection were excluded from the study.

### Sample collection and processing

FNA samples were collected by a pathologist from the affected nodes. Briefly, the swollen area was cleaned with 70% alcohol and then 21 gauge needle was inserted into the mass. After removing the needle, drops of aspirate were placed on a clean slide for FNA cytology, thereafter, leftover FNA samples were added aseptically into sterile universal tubes in phosphate buffer saline pH 7.2 at 4 °C, and transported in cold chain to TB laboratory at Aklilu Lemma Institute of Pathobiology (ALIPB), for further microbiological and molecular analysis.

### FNA concentration for detection of *M.tubeculosis*

FNA specimens were homogenized with 0.85% normal saline and then decontaminated and digested with 4% NaOH for 15 min at room temperature, the samples then concentrated by centrifugation at 3000 g for 15 min at Aklilu Lemma Institute of Pathobiology Mycobacteriology Laboratory [3]. The sediment was neutralized with 2N hydrochloric acid, using phenol red as an indicator. After centrifugation, the supernatant was decanted carefully and 2–3 drops of the sediment were placed on a clean slide and stained with Ziehl-Neelsen (ZN) staining.

### Culture

After homogenization the sediment of processed samples was inoculated onto the conventional Lowenstein-Jensen (LJ) egg slant medium, containing 0.6% sodium pyruvate and glycerol and incubated at 37 °C for at least 6 weeks, with weekly observation for the presence of mycobacterial colonies. Microscopic examination of the colonies was performed using Ziehl-Neelsen stain to select AFB-positive isolates.

### DNA extraction

Mycobacterial genomic DNA was extracted as previously described by Van sooligen et al. [15] with minor modifications. Briefly, 0.2 ml of the FNA material was centrifuged at 1200 rpm for 20 min and the supernatant was discarded. Thereafter the pellet was re-suspended in 500 μl TE. 50 μl of 10 mg/ml of lysozyme (Boehringer Mannheim, Mannheim, Germany) was added and mixed well before incubation for 1 h at 37 °C. The lysozyme-treated samples were incubated at 65 °C for 10 min in the presence of 6 μl of 10 mg of proteinase K/ml (Boehringer Mannheim) and 70 μl of 10% sodium dodecyl sulfate (Boehringer Mannheim, Mannheim, Germany) 0.100 μL of 5 M NaCl and 80 μL of pre-warmed CTAB/NaCl (Cetyl trimethyl ammonium bromide in sodium chloride) (Merck, p.a) solution was added, and vortexed until the liquid content become “milky” followed by incubation at 65 °C for 10 min. Extraction was performed by adding approximately 700–800 μl of chloroform/isoamyl alcohol (24:1) (Merck, p.a) and then centrifuged for 10 min at 12,000 rpm. The aqueous phase was transferred to a fresh micro centrifuge tube. The DNA precipitate was obtained by adding 450 μl of isopropanol (Merck, p.a) to the aqueous phase. After storage for 1 h at −20 °C, the DNA was collected by centrifugation at 12,000 rpm for 15 min, washed with cold 70% ethanol, and centrifuged for 5 min at 12,000 rpm. Most of the supernatant was removed and placed at room temperature for 15 min to evaporate all the ethanol. Finally, the pellet was resuspended in 1xTE and stored at −20°C until the PCR assay was performed.

### Region of difference 9-based deletion typing by PCR

Identification of mycobacterial species causing TBLN was performed using PCR based RD9 and RD4 deletion typing based PCR were used as described earlier [16], using RD9_FlankF, IntR and FlankR and RD4 flankF, IntR and flankR primers. The primers used were RD9_FlankF, IntR and FlankR and RD4 flankF, IntR and flankR primers, and their sequences were published earlier [17]. PCR was performed using a standard thermocycler (VWR Thermo cycler, VWR International, UK). The reaction mixture RD9-based PCR consisted of 10 μl Hot-StarTaqMaster Mix (Qiagen, Crawley, UK), 7.1 μl Qiagen water, 0.3 μl of each of the three primers (100 mM) and 2 μl DNA template giving a total volume of 20 μl. *M. tuberculosis* H37Rv and *M. bovis*, and Qiagen water were used as positive and negative controls, respectively. The reaction mixture was then heated using Thermal Cycler PCR machine (VWR International, UK) using the following amplification program: 95 °C for 10 min for enzyme activation; 95 °C for 1 min for denaturation; 61 °C for 0.5 min for annealing; 72 °C for 2 min for extension, involving 35 cycles all in all; and final extension at 72 °C for 10 min. The product was electrophoresed using the Agarose Gel Electrophoresis System (Bio-Rad, Hernel Hempstead, UK) in 1.5% agarose gel in 1 × EDTA running buffer. Ethidium bromide at a ratio of 1:10, 100 base pair DNA ladder, and orange 6× loading dye were used in gel electrophoresis. The gel was visualized in a MultiImage TM light cabinet using Alpha Innotech, version 1.2.0.1 (Alpha Innotech Corporation, Cannock, UK). The results were interpreted as M. tuberculosis (RD9 present) when a band size of 396 bp was observed and as either *M. bovis* or *M. africanum* (absence of RD9) when the band size of 575 bp was observed. The absence of RD9 was further differentiated by RD4 typing; interpreted as *M. bovis* (RD4 deleted) when the band size of 446 was observed whereas presence of RD4 (*M. tuberculosis* or *M. africanum*) when the band size of 335 was detected.

### Data analysis

All demographic and laboratory data were entered, cleared and analyzed using SPSS version 20 (SPSS Inc., Chicago, IL, USA). The Categorical data was analyzed using the Chai-Square (*X* ^2^) or the fisher exact test that assumes when expected cell size *n* > 5. A *p*-value ≤ 0.05 was considered statistically significant. The confidence level of 95% was used for statistical significance. The criteria for cyto- histopathological diagnosis was epithelioid cell granulomas with or without multinucleate giant cells, with or without necrosis and caseous necrosis without granuloma. Accuracy is defined as the closeness of the measured result to the true value and culture used as a gold standard in our study.

## Result

### Socio-demographic characteristics of the study subjects

A total of 132 clinically suspected TBLN patients who fulfilled the inclusion criteria were included in our study. The study participants’ age ranged from 18 to 71 years old with the mean age of 31.4 years and +/- 12.2 standard deviation. More than half 52.2% (69/132) of presumptive TBLN cases were found in the age group of 18 to 27 years. Out of 132 suspected patients, 55.3% (73/132) were females. The majority 66.6% (88/132) of clients were presumptive TBLN presented with cervical lymphadenopathy. Lymph node condition was reported as hard matted in 47.7% (63/132) of the cases.

### Detection rate

Cytomorphological features of FNAC consistent with tuberculosis lymphadenitis were reported in 83.3% (110/132) of the examined smeared specimens. All FNA samples were processed for bacterial growth on Lowenstein–Jensen (L-J) media, of which 56.1% (74/132) were confirmed as TBLN. According to species identification protocol, no non-tuberculous mycobacterium was reported. *M.tubeculosis* were detected in 39 FNA making the detection rate of direct ZN-smear 29.5% and the detection rate of concentration method of ZN-smear become 65.2%. Thus the concentration methods detect 47 extra patients with an increased yield of 35.6% (47/132) over direct ZN method. The ZN concentration method detection rate was very similar to a molecular method which detects 68.9% (91/132) of FNA samples.

### Accuracy and level of agreement of different diagnostic modalities

The different diagnostic accuracy and level of agreement were compared. Of 74 culture positive FNA 32 (43.2%) and 70 (94.5%), were smear positive on direct ZN staining and on NALC-NaOH concentration methods respectively. Moreover, 69 (93.2%) and 71 (95.4%) showed positive signal on direct PCR and indicated cyto-morphological features consistent with TB respectively. The sensitivity of ZN concentration method and direct PCR was ideal compared measures; however, the sensitivity of direct smear microscopy was less than those methods 43.2%. The level of agreement of concentrated ZN smear microscopy was 0.62 which was ideal compared measures with a kappa of 0.58 of direct PCR in detection of TB against culture. The low sensitivity of direct ZN staining was also reflected in the low 0.32 kappa agreement measure Table 1.

**Table 1:** Efficacy and Kappa values of diagnostic yield of different diagnostic methods used in the diagnosis of TBLN

Diagnostics Methods	Culture			Sensitivity (%)	Specificity (%)	Kappa (95% CI)
		Positive *n* (%)	Negative *n* (%)			
Direct ZN staining	Positive (*n* = 39)	32 (82.1)	7 (17.9)	43.2	87.9	0.32 (0.21–0.41)
	Negative (*n* = 93)	42 (45.2)	51 (54.8)			
Concentrated ZN staining	Positive (*n* = 86)	70 (81.4)	16 (18.6)	94.5	72.4	0.62 (0.57–0.81)
	Negative (*n* = 46)	4 (8.7)	42 (89.3)			
Direct PCR	Positive (*n* = 91)	69 (75.8)	22 (24.2)	93.2	62.1	0.58 (0.41–0.71)
	Negative (*n* = 41)	5 (12.2)	36 (87.8)			
Cytology	Positive (*n* = 110)	71 (64.5)	39 (35.5)	95.4	32.8	0.26 (0.22–0.046)
	Negative (*n* = 22)	3 (13.6)	19 (86.4)			

### Cytomorphological characteristics

A total of 91.7% (121/132) presumptive FNA analyzed consistent the cytological picture of tuberculous lymphadenitis based on the mentioned cytomorphological criteria. Of these, the concentration method and the direct PCR method detect AFB ideal compared measures Table 2.

**Table 2:** Relation of FNAC result on Concentrated and direct ZN smear positivity compared with direct PCR positivity

FNAC result	Total cases % (*N*)	Direct ZN staining		Concentrated ZN staining		Direct PCR	
		Positive % (n/N)	Negative % (n/N)	Positive % (n/N)	Negative % (n/N)	Positive % (n/N)	Negative % (n/N)
TBLN	91.7 (121/132)	32.2 (39/121)	67.8 (82/121)	69.0 (83/121)	31.4 (38/121)	71.9 (87/121)	28.1 (34/121)
Suppurative abscess	3 (4/132)	0.0 (0/4)	100 (4/4)	25.0 (1/4)	75.0 (3/4)	25.0 (1/4)	75.0 (3/4)
Reactive LN	2.3 (3/132)	0.0 (0/3)	100 (3/3)	66.7 (2/3)	33.3 (1/3)	66.7 (2/3)	33.3 (1/3)
Others diagnosis	3 (4/132)	0.0 (0/4)	100 (4/4)	(0.0) 0/4	100 (4/4)	25.0 (1/4)	75 (3/4)

*LN* Lymphadenitis

Cytomorphological consistent of tuberculosis lymphadenitis for all case was clearly described. Of these, the most 42% (56/132) showed epithelioid cell with necrosis and the least cases 11.4% (15/132) were reported in polymorphs with necrosis. Other cytological patterns had shown in. While overall increased AFB positivity rate was observed in necrosis aggregated epithelioid cells, decreased rate was observed in polymorphs cells with necrosis Table 3.

**Table 3:** Cyto-morphological patterns in AFB positivity (concentrated and direct smears) compared with PCR and culture positivity

Cytological patterns	Cases % (n/N)	Direct ZN positive % (n/N)	Concentrated ZN positive % (n/N)	Direct PCR positive % (n/N)	Culture positive % (n/N)
Epithelioid cell with necrosis	42 (56/132)	44.6 (25/56)	73.2 (41/56)	77.0 (42/56)	62.5 (35/56)
Epithelioid cell without necrosis	31.8 (42/132)	9.5 (4/42)	61.9 (26/42)	69.0 (29/42)	66.7 (28/42)
Necrosis without epithelioid cell	14.4 (19/132)	31.6 (6/19)	52.6 (10/19)	63.2 (12/19)	57.9 (11/19)
Polymorphs with necrosis	11.4 (15/132)	20.0 (3/15)	33.3 (5/15)	33.3 (5/15)	66.6 (10/15)

We tried to associate and compared some factors in ZN-staining concentrated method and molecular method. AFB positivity by the concentration method and molecular method was increased in caseous aspirates as compared to purulent and hemorrhagic aspirates though it was not statistically significant (*p*-value = 0.18) and (*p* = 0.62) respectively. However, AFB positivity by the concentration method and molecular method was statistically associated with tenderness of the lymph node (*p*-value = 0.04) and (*p*-value = 0.02) respectively Table 4.

**Table 4:** Factors assessed for associated with positivity when considering concentration and molecular method in Addis Ababa

Variables		Concentration method % (n/N)		OR [95% CI]	*P*-value	PCR method % (n/N)		OR [95% CI]	*P*-value
		Positive	Negative			Positive	Negative		
Sex	Female	63.0 (46/73)	37.0 (27/73)	1	0.6	67.8 (40/59)	32.2 (19/59)	1	0.8
	Male	67.8 (40/59)	32.2 (19/59)	1.2 [0.6–2.5]		69.8 (51/73)	30.1 (22/73)	0.9 [0.4–1.9]	
Age	18–27	65.2 (45/69)	34.8 (24/69)	0.8 [0.3–2.0]		66.7 (46/69)	33.3 (23/69)	0.5 [0.2–1.5]	
	28–37	71.4 (20/28)	28.6 (8/28)	1.9 [0.7–5.0]	0.6	78.6 (22/28)	21.4 (6/28)	1.7 [0.6–4.4]	0.2
	38–47	50.0 (11/22)	50 (11/22)	1.9 [0.214.2]		54.5 (12/22)	45.5 (10/22)	0.7 [0.1–6.7]	
	48–57	50.0 (2/4)	50.0 (2/4)	0.2 [0.03–2.0]		75.0 (3/4)	25.0 (1/4)	0.2 [0.02–2.1]	
	≥58	88.9 (8/9)	11.1 (1/9)	1		88.9 (8/9)	11.1 (1/9)	1	
Resident	Rural	66.1 (39/59)	33.9 (20/59)	1	0.8	69.8 (51/73)	30.2 (22/73)	1	0.8
	Urban	64.4 (47/73)	35.6 (26/73)	0.9 [0.5–1.9]		67.8 (40/59)	32.2 (19/59)	1.1 [0.5–2.3]	
Tenderness of LN	Non-tender	73.2 (48/65)	26.8 (17/65)	1	0.04	78.5 (51/65)	21.5 (14/65)	1	0.02
	Tender	56.7 (38/67)	43.3 (29/67)	0.5 [0.2–0.9]		59.7 (40/67)	40.3 (27/67)	0.4 [0.2–0.8]	
Increasing of LN	Slow	71.9 (23/32)	28.1 (9/32)	1	0.6	81.3 (26/32)	18.7 (6/32)	1	0.03
	Moderate	62.2 (46/74)	37.8 (28/74)	0.7 [0.2–2.3]		68.9 (51/74)	31.1 (23/74)	0.3 [0.1–0.8]	
	Fast	65.4 (17/26)	34.6 (9/26)	1.2 [0.4–2.9]		53.8 (14/26)	46.2 (12/26)	0.5 [0.2–1.3]	
No of LN	Single	74.0 (34/46)	26.0 (12/46)	1	0.3	82.6 (38/46)	17.2 (8/46)	1	0.1
	Few	60.3 (41/68)	39.7 (27/68)	0.5 [0.1–1.8]		61.7 (42/68)	38.2 (26/68)	0.3 [0.1–1.1]	
	Many	61.1 (11/18)	38.9 (7/18)	1.0 [0.3–3.0]		61.1 (11/18)	38.9 (7/18)	0.9 [0.3–2.8]	
Mobility LN	Non-mobile	61.4 (59/96)	38.6 (37/96)	1	0.15	64.6 (62/96)	35.4 (34/96)	1	0.1
	Mobile	75.0 (27/36)	25.0 (9/36)	0.5 [0.2–1.2]		80.5 (29/36)	19.5 (7/36)	0.4 [0.2–1.1]	
Types of aspirate	Purulent	60.0 (49/86)	40 (37/86)	1		63.9 (55/86)	36.1 (31/86	1	
	Caseous	91.9 (34/37)	8.1 (3/37)	1.8 [0.6–11.3]	0.18	83.8 (31/37)	16.2 (6/37)	1.4 [0.4–5.6]	0.62
	Hemorrhagic	33.3 (3/9)	66.7 (6/9)	0.12 [0.1–0.4]		55.5 (5/9)	44.5 (4/9)	03 [0.1–0.9]	

## Discussion

The diagnosis of TBLN has been a true challenge solely by clinical evidence in the developing countries due to the limited diagnostic facility on hand [18]. If the diagnosis of TBLN can be established faster, and the diagnostic process becomes less cumbersome for the patient, PCR may reduce a delay both in the diagnosis and in the start of treatment [19]. But in resource limiting setting like Ethiopia, the method is not feasible. Thus, the method concentrating FNA sample that has similar accuracy with the molecular method but with the low cost required.

The study has shown that the concentration of lymph node aspirate followed by centrifugation was significantly improves the sensitivity of smear microscopy compared measures to the molecular method [20]. The present finding demonstrates comparable result with the study done in Bangladesh [20]. The detection rate of the direct method was 29.5% and it increased to 65.2% in the concentration method which was ideal compared measures to 68.9% in the molecular method. The increased smear positivity by the concentration method which was ideally a comparable measure to PCR was given the evidence to the higher density of bacilli per microscopic field and increase clarity of microscopic field.

The low detection rate and the low sensitivity of direct ZN-staining of the present study was consistent with earlier finding [21]. The low bacilli found in the lymph node aspirates and more than 10,000 bacilli/ml of sample required to be positive on smear microscopy could be the main factor for the decreased sensitivity and low detection rate of direct smear. Though the sensitivity of this method was found to be relatively low, the high specificity of this method allowed to the clinician in of initiation of anti-TB treatment which eliminated antibiotic and anti-TB trials for the patient.

Positivity rate of tuberculosis lymphadenitis was increased in direct and concentrated microscopy and molecular method like PCR when a cytomorphological characteristic of FNAC was epithelioid cell with necrosis aggregates. By the concentration and molecular methods, there is an increase in AFB positivity from 61.9 to 73.2% and 69.0 to 77.0% respectively in those with epithelioid cell aggregates without necrosis to when epithelioid cell aggregates and present with necrosis. Our finding is in line with studies done by others that showed increased positivity for different cytomorphological patterns in smears revealing necrosis aggregates with epithelioid cell [22, 23]. This finding can be explained by the fact that a predominantly necrotic reaction of the tubercle contains more bacilli.

The current study of TBLN showed that the concentration method (91.9%) and on molecular method (83.8%) was high positive yield result as compared to hemorrhagic and purulent aspirates though it didn’t show any significance. This might be due to the fact that the caseous aspirate perforates the deep fascia and escapes into the superficial fascia resulting in collar abscess formation and believed to contain large numbers of bacilli in an advanced stage of the disease [21].

## Conclusion

In conclusion, the low sensitivity of the clinico- microbiological techniques in extra pulmonary specimens have opens the door for the increased use of molecular methods for their high sensitivity and specificity but, their evaluation in low-income countries have been limited. To overcome this problem, the method which has similar sensitivity with PCR should be used. To the effect of this, an increased sensitivity observed in smear concentration method ideally compare measures with PCR can be recommended.

## Abbreviations

CAS: Central Asia
DNA: Deoxy ribonucleic acid
DR: Direct repeat
DST: Drug susceptibility test
ETH: Ethiopia
FNA: Fine needle aspirate
INH: Isoniazid
LJ: Lowenstein-Jensen
LPA: Line probe assay
MDR-TB: Multi-drug resistance tuberculosis
PCR: Polymerase chain reaction
RMP: Rifampin
RNA: Ribonucleic acid
SIT: Spoligo international type
TB: Tuberculosis
TBLN: Tuberculosis lymphadenitis
WHO: World Health Organization
WT: Wild type
